# Limited Evidence to Review—Is There an Association Between Cognition and Upper Extremity Motor Reaction Time in Older Adults?

**DOI:** 10.3390/neurosci6030071

**Published:** 2025-07-30

**Authors:** Alexandria Jones, Natalie Weaver, Mardon E. So, Abbis Jaffri, Rosalind L. Heckman

**Affiliations:** 1 School of Medicine, Creighton University, Omaha, NE 68178, USA; alliejones@creighton.edu; 2 Department of Physical Therapy, Creighton University, Omaha, NE 68178, USAabbisjaffri@creighton.edu (A.J.)

**Keywords:** older adult, cognition, reaction time, sensorimotor control, age

## Abstract

Optimal sensorimotor control depends on response timing. With age, it is broadly assumed that reaction time (RT) increases as cognitive function declines. However, it is not clear if the literature supports this assumption. The purpose of this work was to review the association between cognition and upper extremity RT in older adults. We conducted a search using Scopus database with four inclusion criteria: (1) healthy, community-dwelling adults over 60 years old, (2) upper extremity movement, (3) cognitive assessment, and (4) RT measure. Twenty-five of the 1608 articles screened met the inclusion criteria. Only nine studies directly or indirectly assessed the association between cognition and RT. Our interpretation of the literature was further limited by inconsistency in test selection and measurement interdependence that could be addressed by future studies. We present a conceptual framework to guide research assessing the influence of cognition on sensorimotor control with age.

## 1. Introduction

Response timing is essential to optimal sensorimotor control across the lifespan. Daily activities, such as extending the arm to answer the phone, reach a glass of water, or recover balance, require a complex interplay of cognitive and motor function to generate timely responses to environmental stimuli [1]. One measure of the timing of sensorimotor responses is the reaction time (RT), or the measure of time from an external stimulus to the onset of a motor response. RT literature has traditionally been used to investigate motor preparation as a prerequisite to movement execution [2,3,4], though emerging literature has shown how preparation and execution can overlap in time [5,6]. RT has been used to gauge the healthy interplay between cognitive and motor function, and it has been recognized that cognition influences motor function through decision-making [7], competition [8], and difficulty [9]. It is conceivable that the interplay between cognitive and motor function occurs during motor preparation. For example, the selection of a movement goal [10] depends on attention, sensation and perception, and executive function, which typify domains of cognitive function [11,12,13]. However, evidence that aging [14] and cognitive reserve [15] have differential effects on motor preparation and performance suggests the relationships between age, cognition, and motor function are complex and may differ from expected trends [16]. It is unknown to what extent the literature supports a general assumption that sensorimotor RTs increase as cognitive function declines in older adults.

RTs measured in older adults are longer [17,18], but longer RTs have not been consistently associated with measured declines in either global cognition [19,20] or specific domains of cognitive function [21,22,23,24]. Longer RTs have been observed in older adults for upper extremity movements of the arm [25], hand [26,27,28] and finger [29]. These longer RTs may result from (1) delayed movement initiation [30,31] or (2) increased time required to prepare the desired movement [25,32,33,34]. The hypothesis that longer RTs result from delayed movement initiation is supported by decision threshold models [30] suggesting that older adults have higher decision thresholds, which demand more evidence, thus more time, to initiate a motor response compared to younger adults. However, even with longer RTs, older adults have decreased movement accuracy in response to temporally unpredictable stimuli (whereas the predictable cue counteracts the age-related increase in RT) [34]. A recent study experimentally dissociated movement preparation and initiation [35,36,37] and found empirical evidence for the hypothesis that longer motor preparation times contributed to longer RTs in older adults [25]. Since increased motor preparation time contributes to longer RTs, we suggest that declines in specific cognitive domains with the potential to influence preparation could be associated with declining motor function in older adults. This suggested relationship has been supported by prior research in a learning task that showed deficits in two cognitive domains, executive function and learning and memory, were associated with decreased accuracy in predicting the association between a stimulus cue and outcome [38]. However, it is not known whether deficits in specific cognitive domains are associated with longer RTs in older adults in motor, rather than learning, tasks.

The purpose of this narrative review was to determine the association between specific cognitive domains and upper extremity motor function in older adults. Upper extremity function was selected because of its role in daily activities and interactions with the environment, as well as its prominence in sensorimotor control [39,40] and rehabilitation [41] literature. RTs traditionally used to investigate sensorimotor control are used as a measure of motor function in this review. Sensation and perception, motor skills and construction, perceptual motor function, executive function, attention, learning and memory, and language are specific domains of cognition considered along with global measures of cognitive function because of their role in identifying cognitive function for diagnosis across the continuum of neurocognitive disorders [11,12].

A narrative review was selected to synthesize the literature on the association between cognition and upper extremity motor RT in older adults due to the broad and interdisciplinary nature of the topic relevant to basic science, medicine, clinical research, and industry. Unlike systematic reviews, which require narrowly defined research questions and strict inclusion criteria, or meta-analyses, which necessitate homogeneous studies for quantitative synthesis, a narrative review allows for a flexible and interpretive synthesis of diverse study designs, methodologies, and cognitive domains. This approach was deemed appropriate given the limited number of studies directly associating cognitive domains with RT measures and the heterogeneity of cognitive and motor assessments in the literature, which precluded a meta-analytic approach. This narrative review provides syntheses of literature evaluating cognitive function and RT in upper extremity movements in community-dwelling adults over 60 years of age, which has never been studied, and provides a scaffold for future original research projects and subsequent systematic reviews.

## 2. Materials and Methods

We conducted a search for this narrative review using the Scopus database, and then manual searches of the citations were undertaken. The search included all literature published in the most recent 10 years up to the date of the search. The following search strategy was used:

“Old individuals” OR “geriatrics” OR “older adults” AND “cognitive decline” OR “cognitive deficits” OR “cognitive deficit” OR “cognitive impairment” OR “executive function” OR “complex attention” OR “memory impairments” OR “sensory perceptual impairment” AND “motor function” OR “motor deficits” OR “reaction time” OR “motor impairment” OR “motor dysfunction”.

The Scopus database was selected due to its comprehensive coverage of peer-reviewed literature across disciplines relevant to this review, including cognitive neuroscience, motor control, gerontology, and rehabilitation medicine. Scopus was selected for its robust indexing, which ensured access to high-quality, peer-reviewed publications, and advanced search functionalities, which facilitated the identification of articles aligned with the review’s objectives. Use of the Scopus database for the literature search provided a representative sample of the literature that balanced comprehensiveness with practical constraints such as resource availability and overlap with other databases.

**Figure 1:**
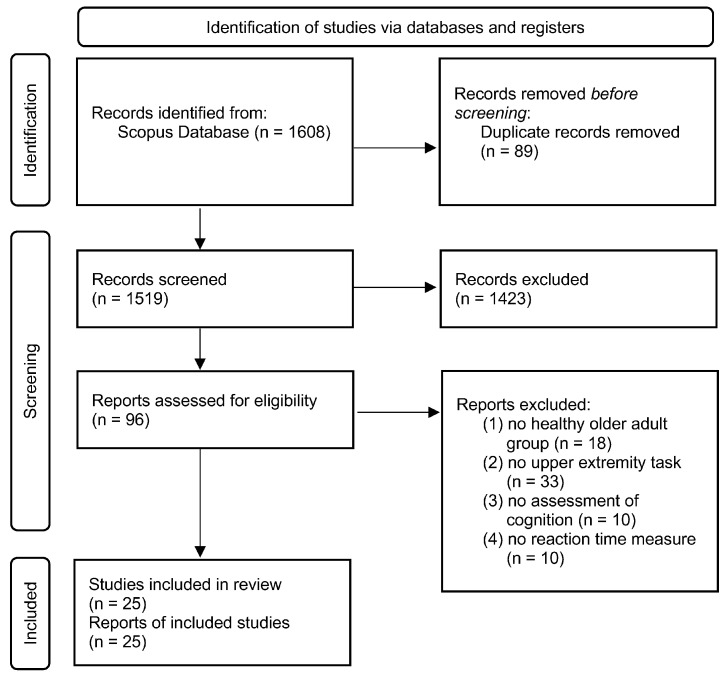
PRISMA-style flowchart of study inclusion [43].

The initial search was completed on 14 June 2022. After searching the database and removing the duplicates, literature screening and data extraction were performed. Four reviewers independently screened the titles. Discrepancies in screening were resolved through discussion and consensus was reached for all studies. The search was updated on 15 August 2024 and 10 May 2025, with two reviewers independently screening the additional titles. Relevant abstracts and subsequent full-length articles were screened based on inclusion criteria (Figure 1). Systematic reviews, meta-analyses, protocol publications, and studies not written in English were excluded. The articles included in this review are based on the following inclusion criteria:

(1) The study population included healthy, community-dwelling older adults (age > 60 years)
(2) A physical upper extremity movement task was performed (examples include reaching, finger tapping, and button press tasks)
(3) At least one measure of cognitive assessment was performed
(4) An RT measure was quantified from the upper extremity task

To assess the methodological quality of the included studies, articles were scored using the Physiotherapy Evidence Database (PEDro) scale [42]. Two reviewers scored the articles independently, and a third reviewer arbitrated discrepant scores until consensus was reached (Table 1).

**Table 1:** PEDro scale criteria for reviewed articles. Criteria 1–11 were scored “yes” if the criterion was met by the study. Points for each “yes” were summed for total PEDro scores according to https://pedro.org.au/english/resources/pedro-scale/ (accessed on 24 October 2022).

PEDro Criteria	Study																								
	Bao et al. (2019) [44]	Chen et al. (2020) [45]	Ferreira et al. (2022) [46]	Hartle et al. (2022) [47]	Hennessy et al. (2025) [48]	Hong et al. (2020) [49]	Jardim et al. (2024) [50]	Juhasz et al. (2019) [51]	Jutten et al. (2023) [52]	Kimura et al. (2023) [53]	Kitchen and Miall (2019) [54]	Korthauer et al. (2019) [55]	Krumpolt et al. (2025) [56]	Mack et al. (2025) [57]	Qiu and Xiong (2017) [58]	Rattanavichit et al. (2022) [59]	Sleimen-Malkoun et al. (2013) [60]	Staub et al. (2014) [61]	Tait et al. (2024) [62]	Unger et al. (2025) [63]	Van Humbeeck et al. (2024) [64]	Vasquez et al. (2016) [65]	Welhaf et al. (2024) [66]	Worschech et al. (2024) [67]	Yao et al. (2016) [68]
1	yes	yes	yes	yes	yes	yes	yes	yes	yes	yes	yes	yes	yes	yes	yes	yes	yes	no	yes	yes	yes	yes	yes	yes	yes
2	no	no	yes	no	no	no	no	no	no	no	no	no	no	yes	no	no	no	no	yes	no	no	no	no	no	no
3	no	no	no	no	no	no	no	no	no	no	no	no	no	yes	no	no	no	no	yes	no	no	no	no	no	no
4	no	yes	yes	yes	no	no	yes	no	yes	yes	yes	yes	no	yes	no	yes	no	no	yes	no	no	no	yes	yes	yes
5	no	no	no	no	no	no	no	no	no	no	no	no	no	yes	no	no	no	no	yes	no	no	no	no	no	no
6	no	no	no	no	no	no	no	no	no	no	no	no	no	no	no	no	no	no	yes	no	no	no	no	no	no
7	no	no	no	no	no	no	no	no	no	no	no	no	no	no	no	no	no	no	no	no	no	no	no	no	no
8	yes	yes	yes	yes	yes	yes	yes	yes	yes	yes	yes	yes	yes	yes	yes	yes	yes	yes	no	yes	yes	yes	yes	yes	yes
9	no	no	yes	no	no	no	no	no	no	no	no	no	yes	yes	no	no	no	no	yes	yes	no	no	no	no	no
10	yes	yes	yes	yes	yes	yes	yes	yes	yes	yes	yes	yes	no	yes	yes	yes	yes	yes	yes	yes	yes	yes	yes	no	no
11	yes	yes	yes	yes	yes	yes	yes	yes	yes	yes	yes	no	yes	yes	yes	yes	yes	yes	yes	yes	yes	yes	yes	yes	no
Score	4	5	7	5	4	4	5	4	5	5	5	4	4	9	4	5	4	3	9	5	4	4	5	4	3

PEDro: Physiotherapy Evidence Database.

Measures of RT were included for simple, choice, or complex movement tasks. Simple RT tasks are those that have a known movement that can be prepared in advance of a stimulus cuing movement initiation [2,3,4]. Choice RT tasks have multiple possible movement goals, and the desired movement may be cued by the stimulus for movement initiation. Complex RT tasks do not meet the criteria for simple or choice RT tasks and often have distracting information or require decision-making in response to the movement stimulus.

Measures of cognitive function were included from cognitive assessments for either global cognition or specific cognitive domains. We considered seven specific cognitive domains, often measured through a complete neuropsychological battery, based on integration of the domains of cognitive function used in the literature and for clinical diagnosis [11,12]. The specific cognitive domains were sensation and perception, motor skills and construction, perceptual motor function, executive function, attention, learning and memory, and language.

Since our purpose was to review the literature for the association between cognitive and motor declines in older adults, we considered the association between independent measures of cognitive and motor function. That is, we reviewed studies with a cognitive assessment that did not depend on RT and with an RT measure from a simple task that did not depend on cognition. To help elucidate the challenges of quantifying simple motor RTs and specific measures of cognition, we also considered studies that used an RT task as a measure of cognitive function or studies that measured RT from choice or complex tasks influenced by cognitive function.

## 3. Results

Twenty-five articles met the study inclusion criteria, with an increase in published studies from 2022–2025. The methodological quality of most articles was fair (PEDro scores: 4–5) and ranged from poor to excellent (PEDro scores: 0–3 and 9–10, respectively).

Upper extremity movement tasks frequently included a physical or touch screen button press, key press, or mouse click. Eleven articles measured both a cognitive assessment independent of motor function and a simple RT [46,47,48,50,52,56,58,59,60,62,64]. Fourteen articles studied a choice or complex motor RT task, thus engaging cognitive processes beyond simple motor RT performance [44,45,49,51,53,54,55,57,61,63,65,66,67,68].

The association between cognitive function and motor RTs has not been widely reported. Only five studies directly assessed the relationship between measures of cognition and RT (Table 2A). An additional four studies indirectly assessed this relationship. In these studies, associations were assessed for cognitive and RT measures that were interdependent [44,51] or combined across sessions [52] or groups [65] (Table 2B). The majority of the reviewed studies measured both cognition and RT but did not analyze their association (Table S1).

Eleven articles [46,47,48,50,52,56,58,59,60,62,64] measured both a cognitive assessment independent of motor function and a simple RT (Table 2A). Upper extremity tasks frequently included a physical or touch screen button press, key press, or mouse click. The cognitive domains most often assessed included perceptual motor function [59], especially the subdomain of processing speed [46,47,48,52,58,60,62], executive function [52,56,58,59,62], and learning and memory [47,48,50,52,62,64]. Motor skills and construction [47,48,60], attention [47,48,50], and language [46,47] domains were also assessed. The sensation and perception domain was not assessed. Zero reviewed studies assessed the association between cognition and simple RT. One study assessed the association between cognition and a combined measure of simple and choice RT and found variability between sessions across a one-year period was associated with global cognition, processing speed, and memory [52].

We found an interdependence of cognitive and motor processes was evident for cognitive domains of processing speed [47], attention [61], and learning [49,51,67]. This interdependence was characterized by cognitive domain measures derived from motor RT tasks [49,51,61] including two studies that only assessed cognition using measures dependent on motor RT [44,51]. An interdependence of cognitive and motor processes was also evident in studies of dual performance of cognitive and motor tasks resulting in longer RTs [44,64] and poorer cognitive performance [44].

The strength of evidence for an association between global cognition and specific cognitive domains and subdomains was limited (Table 3). Global cognition was assessed in multiple reviewed studies [53,55,65] and used as inclusion or exclusion criteria [45,49,54,68] but only compared to RT measures in one study [53]. One study assessed the association between cognition and a combined measure of simple and choice RT and found variability between sessions across a one-year period was associated with global cognition as well as processing speed and memory [52]. Two cognitive domains, sensation and perception and motor skills and construction, were infrequently measured and reported in the reviewed articles [47,48,49,54,60,67]. The perceptual motor function domain was assessed in several studies. Across the reviewed articles, the processing or perceptual speed subdomain of perceptual motor function was associated with variability in RT in both choice [66,68] and complex movement tasks [53,68]. Visuospatial attention, which may be relevant to both perceptual motor function [12] and attention cognitive domains, was measured in one study [55] and challenged by a motor task in another study [49], but the relationship between visuospatial attention and RT was not assessed independently. The executive function domain was assessed in several studies and associated with choice [55,68] and complex [65,68] RT measures. The attention domain was assessed by cognitive tests also used to measure processing speed (e.g., [48,53]) and by sustained and inhibitory tests (e.g., [47]), though infrequently assessed for an association with RT [53,66]. One study measured preparatory attention using electroencephalographic recordings of neural oscillations and correlated the relationship between neural recordings and RT with cognitive domain measures [45]. The learning and memory domain was assessed in several studies. The memory subdomain was associated with variability in choice [55,66,68] and complex [65,68] RT measures. Decreased memory was also associated with increased variability in choice RT when measured longitudinally over multiple years [68]. The learning subdomain has not been directly assessed in relation to motor RT but has been measured as a function of motor performance in a complex movement task [49]. The learning subdomain was not associated with motor RT in older adults when measured as a change in motor function with repeated exposure to a task [51]. For the language cognitive domain, there was mixed evidence for an association with RT [53,65,66,68] though not all studies assessed this association [46,47].

## 4. Discussion

This narrative review considered original studies investigating the association between cognitive function and upper extremity motor function in older adults. The findings indicate limited research has directly associated measures of cognitive domains with RT. We considered measures for either global cognition or specific cognitive domains, including sensation and perception, motor skills and construction, perceptual motor function, executive function, attention, learning and memory, and language. We considered simple RTs to be an optimal measure of motor function and additionally reviewed studies with choice or complex RTs. We used a structured search process to extract relevant studies. We found that the association between simple RT and cognitive function, either global or specific domains, has not been directly assessed. The processing speed subdomain of perceptual motor function, executive function, attention, and the memory subdomain of learning and memory were associated with complex and choice RT or variability in RT measures. There was mixed evidence for an association between the learning subdomain of learning and memory or the language domain and RT in older adults. We found limited evidence assessing the association between global cognition or cognitive domains of sensation and perception, motor skills and construction, and RT. We additionally found that some cognitive tests, for example, Trail Making Tests, digit span, and fluency, were measured to quantify function in more than one cognitive domain. Interest in the relationship between cognitive and motor function in older adults is evident in contemporary literature, and further research is needed to directly assess this relationship.

### 4.1. Executive Function and Memory Are Associated with Choice and Complex RTs

Age-related constraints in executive function have been shown to affect motor planning [69], which may contribute to the importance of executive function for fall risk in older adults with cognitive impairment [70,71,72] and its association with both choice [55,68] and complex [65,68] RT measures. Further, executive function has been associated with use of internal memory strategies in older adults [73], though compensatory use of external memory strategies typically occurs with age [74]. In the reviewed studies, RT differences in older adults were associated with declines in executive function measured using components of a neuropsychological battery or other cognitive assessments. In addition, longer RTs were associated with declines in working memory measured as executive function. Additional components of the memory subdomain were generally, though not always, associated with RT in older adults.

While four reviewed studies have suggested an association between both executive function and memory domains and upper extremity motor function in older adults, knowledge of the influence on simple motor RT as a basis for sensorimotor control is limited when using choice and complex RT measures. Choice RT measures in the reviewed studies required participants to select the motor goal from one of multiple targets with both internal [55] and external [55,68] representations of uncertainty. Korthauer and colleagues found the association between cognitive function and RT was affected by the representation of uncertainty [55], which suggests cognitive domains can independently influence sensorimotor control. For internally-driven uncertainty, poor performance in the executive function domain was associated with longer RTs, and for externally cued uncertainty, poor performance in the memory domain was associated with longer RTs [55]. Yao and colleagues also used an experimental task with externally-cued uncertainty, and their results showed decreased performance in both executive function and memory domains were associated with greater variability in RT in older adults. Further, decreased performance in the memory domain during the four-year study period was associated with increased variability in choice RT [68]. Complex RT measures in the reviewed studies required participants to process dynamic sensory input [65] or remember a stimulus feature or prior cue [52,68]. Similar to findings for choice RT tasks, performance in executive function and memory were associated with greater RT variability in complex RT tasks [65,68]. Jutten and colleagues measured simple, choice, and complex RTs monthly over one year and found that greater variability in combined simple and choice RT was associated with poorer baseline performance in memory, while greater variability in complex RT was associated with poorer baseline performance in executive function [52]. The two tasks measured for complex RT [52] have also been used as measures of executive function and learning and memory domains [62]. The complex nature of motor tasks imposes greater demands for cognitive function, which may contribute to longer RTs. Simple motor RT measures would clarify the role of executive function and memory in sensorimotor control.

### 4.2. The Interplay Between Cognitive and Motor Function Contributes to Interdependence of Select Measures

Cognitive and motor function both contribute to sensorimotor control, and their interdependence may confound typical measures and limit understanding of changes with age. For example, motor function has been measured with the concurrent performance of a cognitive task often referred to as a dual task. A dual task design has been widely used to investigate balance and gait in older adults [75,76,77,78], and some evidence has shown greater motor deficits with dual task paradigms challenging memory [79] or executive function [80,81]. RTs in upper extremity finger and thumb motor tasks were increased with dual task paradigms challenging visuospatial working memory [64] and attention [44]. Bao and colleagues proposed that several factors, including age-related decrease in neural conduction rates, decreased cognitive and motor function, and diminished sensory function, could contribute to RT differences in older adults with and without simultaneous performance of a cognitive dual task but concluded it was not possible to identify a primary factor [44]. Other research supports the contribution of multiple factors to RT differences. A randomized controlled trial with 12 weeks of strength, balance, and cognitive training found improvements in simple RT measures as well as cognitive measures of attention and executive function and motor measures of physical performance and falls [82]. Independent measurement of cognitive and motor function is necessary to determine if baseline changes in cognitive function contribute to decreased motor performance in older adults, or if the cognitive task affects the preparation and/or initiation of the motor task to result in longer RTs.

Cognitive function, in particular the learning subdomain of learning and memory, has not been directly assessed in relation to motor RT; rather, learning has been measured based on changes in motor performance [49,51]. Two reviewed studies used sequence learning tasks and reported longer RTs in older adults with [49] and without [51] cognitive impairment compared to younger adults. Both studies found no association between the cognitive assessment of learning and motor RT in older adults [49,51], but the assessment of learning was dependent on motor performance. In other contexts, research has shown that older adults learn to detect valid task cues as well as younger adults [83], and explicit instructions enhance learning consolidation for complex motor tasks [84]. Motor learning mechanisms [85] and task-related differences in learning [86] are important to consider along with the influence of other cognitive function domains to facilitate optimal sensorimotor control in aging adults.

The interplay between cognitive and motor function is also evident in measures of processing speed. Processing or perceptual speed, named for consistency with the task used to assess it, can be considered as a component of the complex attention domain [12], a separate cognitive domain [11], or a subdomain of perceptual motor function (this review). Variability in simple and choice RT has been associated with poor function in tests of processing speed [52,53]. Though, more often RTs measured from visual-perceptual or visual-motor tasks have been used to assess processing speed. Task difficulty has been systematically varied to show slower processing speeds in older adults [58] affects cognitive and motor neural resources [60]. However, it is not easy to dissociate the speed of cognitive and motor processes when measures depend on the relationship between task difficulty and response timing. That is because motor processing speed depends on sensory processing and knowledge of complex kinematics, while cognitive processing speed depends on motor function known to be influenced by task certainty.

### 4.3. Advancing the Field of Sensorimotor Control for Older Adults

We suggest advances in the understanding of sensorimotor deficits in aging adults could arise from measurements in four areas: global cognition, sensory and perception, motor skills and construction, and RT. Knowledge of the relationship between global measures, specific domains of cognition, and motor function could inform screening and intervention in aging adults by differentiating physiological versus pathological changes.

Traditional assessments of global cognition are typically validated for use in specific populations or during certain healthcare encounters and may measure specific subdomains of cognition. A recent review evaluated the relationship between cognitive function and hand motor function, including grip strength and dexterity [87]. The authors found that hand grip strength was associated with global cognition, frequently measured by the MMSE, in older adults. Hand grip strength was further associated with longitudinal declines in global cognition and specific cognitive domains, including memory, visuospatial ability, and perceptual speed. Hand dexterity, in contrast, was associated with executive function in older adults [87]. The review on hand motor function highlights the complexity of the relationship between global cognition, cognitive domains, and motor function.

The specific cognitive domain of sensation and perception has not been commonly assessed in studies associating cognitive and motor function in older adults, though sensation, the detection of sensory stimuli, and perception, the processing and integration of sensation [11], may be influenced by age. The available research has shown decreased sensitivity to tactile or haptic stimuli with age [88], but differences in perception of force applied at the hand were not explained by proprioception ability as measured by a position matching task [89]. Further, the perception of sensory information and integration into sensorimotor control is dependent on movement, and suppression of tactile sensation from the upper extremity occurs during the planning and execution of reaching movements, as reviewed by Juravle and colleagues [90]. Greater attenuation [91] of vibrotactile sensation was measured with simultaneous performance of a secondary cognitive task and associated with decreased executive function ability in both young and old adults [92]. In this review, one study assessed the sensation and perception domain using the Benton Judgment of Line Orientation [49] as a measure of visuospatial perception [93,94]. Another study measured proprioception from errors in movement accuracy [54], which is a common approach to measuring proprioception along with clinical discrimination tests. Though proprioception training has been shown to improve proprioceptive function [95] and influence the specification of motor commands during preparation [96], it is not known if there is a direct influence on RT.

The specific cognitive domain of motor skills and construction has also not been commonly assessed in studies associating cognitive and motor function in older adults. RT has been included as a measure of motor skill, while motor construction has typically been assessed with copying or drawing tests; for example, the Clock Drawing Test (CDT) [11] without assessing for an association with RT. The CDT has emerged as a widely used neuropsychological assessment or cognitive screen. Despite many formats for administration, including free draw or copy and different times for the clock hands [97], a recent scoping review of cognitive tools found that the CDT was reliable, easy and quick to administer, and among the most frequent cognitive tools used to detect mild cognitive impairment [98]. Historically, the CDT emerged in the assessment of individuals with speech and language disorders, known as aphasia and apraxia, defined by the inability to copy, draw, or otherwise construct despite intact sensation, perception, and motor function (see Hazan and colleagues for review [99]). Though widely used to assess parietal lobe function and motor planning, older adult performance on the CDT has also been correlated with performance in other cognitive domains, including processing speed, working memory, and language [97]. Indeed, emerging evidence for neural activity beyond the parietal lobes during the CDT [100,101,102], may support its use for assessment of both global cognition and motor construction in aging adults.

RT measures have been used to understand both motor and cognitive function but may be limited by analytical approaches. RT has been correlated with significant outcomes, including mortality [103,104,105], in older adults and can be influenced by changes in muscle tissue and function [106,107,108] and in central or peripheral neural drive to the muscle [18,106]. RT has been used as a measure of cognitive function along with other motor skills, such as manual dexterity and balance [11], and cognitive load can be varied through choice and complex motor tasks used to measure RT. Though language varies across fields and authors, the influence of cognition on prolonged RTs can be described in terms of stimulus reception and integration, decision-making to select a desired motor goal, preparation of a motor action, and descending neural drive to achieve muscle activation [10,18,109,110]. Many analytical approaches to RT measures assume the RT is composed of discrete periods, such as processing speed or task switching, that sum to contribute to the overall time. This assumption is present in neuropsychological tests that compare Trail Making Test B and A to measure executive functioning versus processing speed and in decades of studies on motor planning [2,3,111,112,113]. However, this assumption has not been upheld by recent work in upper extremity reaching movements for older adults [25] nor in primate neurophysiology suggesting motor preparation and initiation can overlap temporally [5] and are distributed across neural networks [114,115]. Advanced research approaches are needed to understand the complex relationship of RT measures to both cognitive and motor function.

### 4.4. Limitations

While this narrative review is an important step towards quantifying the relationship between changes in cognitive and motor function with age, thoughtful consideration is due to address study limitations. First, though we employed a systematic search process to inform our narrative review, only a small number of studies met the inclusion criteria. Of the studies that met the inclusion criteria, the majority did not assess the association between cognitive tests and measured RTs. Second, the methodological quality of the reviewed studies was predominantly fair as assessed using the PEDro scale. We synthesized across studies when possible to minimize bias. Third, confounding variables (e.g., sex, education) may have impacted the quality of reviewed studies if not addressed by inclusion criteria, participant grouping, or measurement adjustment. Fourth, there was substantial inconsistency in the selection of cognitive tests and RT measures. Some cognitive tests were used to assess function in more than one domain, and others were only measured by a single study. Similarly, RT measures varied in terms of the movement, task cues, complexity, and quantification. Without standardization of measurement constructs, methodological inconsistencies across studies limit broad interpretation and generalizability.

## 5. Conclusions

**Figure 2:**
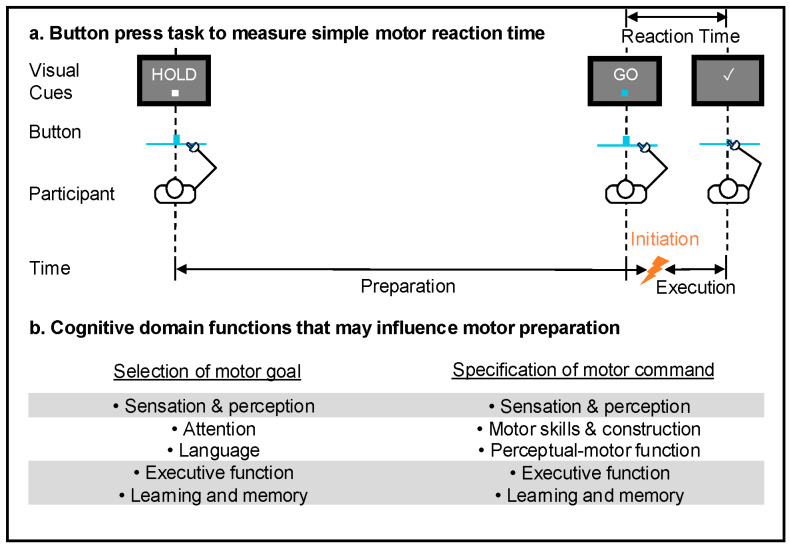
(**a**) Schematic of simple motor RT task with timing of motor preparation, initiation, execution, and RT and (**b**) conceptualization of cognitive domains that may influence motor preparation using terminology from [10,11,12].

The purpose of this narrative review was to determine the association between cognitive function and upper extremity motor RT in older adults. We conducted a systematically informed narrative review and found a lack of published studies that assessed the association between measures for global cognition or specific cognitive domains and measures for simple, choice, or complex RT. Across studies that either directly or indirectly assessed the association between cognitive function and RT, there was an association of poorer cognitive performance in processing speed, executive function, attention, and memory with increased variability in choice and complex RT. Our review is limited by the lack of published studies that associated cognitive and motor measures in older adults. Knowledge gained from published studies was limited by the fair methodological quality of the reviewed studies and inconsistent use of cognitive measures and RT measures beyond simple sensorimotor control. Original research that addresses these limitations is needed to support our understanding of longer RTs measured in aging adults and the influence of cognitive function. We propose a conceptual framework to support elucidation of the relationship between cognitive and motor function with age. Our model of simple RT considers the influence of specific domains of cognition during motor preparation (Figure 2). Future work that includes a direct association between independent cognitive and motor measures would inform the design and selection of screening and intervention tools across engineering and health science disciplines to improve the aging experience.

## Figures and Tables

**Table 2:** Studies that assessed the association between cognitive function and RTs in older adults.

A. Studies that Directly Assessed an Association				
Study Population	Inclusion and Exclusion Criteria	Cognitive Domain or Subdomain [test]	Movement and RT Measures	Results and Associations
Kimura et al. (2023) [53]				
72 ± 3 years *n* = 31 ^∧^ 77% male	Inclusion No diagnosis of MCI or dementia Clinical DRS = 0 Able to perform ADLs and IADLs MoCA ≥ 26 (Japanese version) Exclusion Diagnosis of dementia, MCI, mental disorder, cerebrovascular disease	Global [MoCA] Processing Speed [TMT-A, TMT-B] Executive Function ^a^ [TMT-B] Attention [TMT-A] Language ^b^ [Verbal Fluency Test]	**Right thumb button press** Complex RT Go/No-go task Feature: stimulus letter Frequency: 75% Go, 25% No-go	Intraindividual variability in RT in older adults was associated with poorer performance in global cognition and cognitive domains of processing speed, attention, and language. Intraindividual variability in RT was not associated with a working memory measure of executive function. There were no age-related differences in adjusted intraindividual variability in RT.
Korthauer et al. (2019) [55]				
**Externally cued task** 77 ± 6 years *n* = 12 ^∧^ 25% male **Internally driven task** 76 ± 5 years *n* = 14 ^∧^ 28% male	Inclusion Cognitively healthy Exclusion History of alcohol use History of substance use Learning disabilities Serious psychiatric illness	Global [MMSE, BDS, DRS] Processing Speed [WAIS-R (Digit Symbol Modalities), TMT-A] Visuospatial Ability [WAIS-R (Block Design)] Executive function [TMT-B, Letter Fluency, modified WCST, Clock Drawing Test] Attention ^c^ [WAIS-R (Digit Span)] Learning and Memory [CVLT, WMS-R (Visual Reproduction and Logical Memory)] Language [BNT, WAIS-R (Vocabulary and Category Fluency)]	**Card sorting, externally cued uncertainty** Choice RT 2–5 choices Feature: shape, number of sorting piles cued the stimulus-response uncertainty **Card sorting, internally driven uncertainty** Choice RT 5 choices Feature: shape, relative frequency of shapes varied but was not cued by the number of sorting piles	For the externally cued uncertainty task, longer RTs were associated with poorer memory in the highest uncertainty condition. For the internally driven uncertainty task, longer RTs were associated with poorer executive function. The associations of global cognition, processing speed, attention, and language with RT were not assessed.
Staub et al. (2014) [61]				
65 years (60–74) *n* = 30 ^∧^ 47% male	Inclusion No neurological or psychiatric disease Normal or corrected-to-normal vision	Attention ^d^ [Sustained Attention to Response Task]	**Key press** Complex RT Digits 1–9 presented on screen Target: “3” Response Task: press key only for target Response Inhibition Task: press key for all digits except target	Attention deficits in the response task for older adults were suggested as error rates and RTs increased over time. In the response inhibition task, no deficits in attention were found for older adults as error rates decreased over time while RTs increased.
Welhaf et al. (2024) [66]				
76 ± 6 years (62–76) *n* = 345 ^∧^ 41% male	Inclusion Clinical Dementia Rating = 0	Processing Speed [Digit Span Forward, Number Symbol Test] Attention [Stroop Incongruent, CVOE Switch] Learning and Memory ^e^ [WMS Paired Associative Recall, Free and Cued Selective Reminding Test, Craft Story 21 (Immediate and Delayed Recall)] Language ^f^ [CVFT, Multilingual Naming Test]	**Touch screen tap** Ambulatory Research in Cognition Symbols task Choice RT 2 choices Feature: shape pairs	Greater RT variability was correlated with poorer performance in processing speed, attention, episodic memory, and semantic memory.
Yao et al. (2016) [68]				
74 ± 6 years (64–92) *n* = 304 32% male	Inclusion Older adults concerned about cognitive function Exclusion Diagnosis of dementia or MMSE < 24 History of significant head injury Neurological or major medical illnesses Severe sensory impairment Substance or alcohol use Psychiatric diagnosis Psychotropic drug use Not fluent in English	Perceptual Speed [TMT-A, TMT-B] Executive Function ^g^ [WAIS-III] Memory ^e^ [Immediate free recall] Language [Vocabulary]	**Key press** Choice RT 4 choices Feature: location Choice/Complex RT 4 choices Feature: location of previous cue Complex RT Stimuli choices varied in shape (square, circle) and color (red, green) Stimulus feature cued at beginning of trial	Greater RT variability in choice and complex motor tasks was associated with poorer cognitive function in perceptual speed, executive function, and memory. RT variability was not associated with language ability. Over a five-year period, memory function was associated with decreased RT in choice tasks, while both perceptual speed and memory function were associated with decreased RT in complex tasks.
B. Studies that indirectly assessed an association				
Study Population	Inclusion and Exclusion Criteria	Cognitive Domain or Subdomain [test]	Movement and RT Measures	Results and Associations
Bao et al. (2019) [44]				
71 ± 6 years *n* = 9 44% male	Inclusion No neurological conditions No joint replacements Stand independently > 1 min Exclusion Impaired sensation on the dorsal aspect of the dominant foot	Attention [Backwards counting by 3s]	**Thumb trigger press** Complex RT Respond to vibrotactile stimuli while performing cognitive task	Attention was divided with a dual task paradigm and affected both RT and cognitive performance. • RTs were longer in older adults when attention was divided. Performance speed on the cognitive test decreased with simultaneous performance of the motor task.
Juhasz et al. (2019) [51]				
66 ± 6 years (61–85) *n* = 26 ^∧^ 19% male	Inclusion None Exclusion Participants with response time or accuracy < 3 standard deviations of the group Developmental, psychiatric, or neurological disorders	Learning [General skill learning, triplet learning]	**Key press** Complex RT Alternating serial RT 4 choices Feature: location, frequency of repeated key responses	General skill learning measured by the change in RT with task performance was correlated with average RT in older adults. This correlation was decreased when general skill learning was normalized by the average RT. Triplet learning, measured by the change in RT for low- and high-frequency key press triplets, was not correlated with average RT in older adults.
Jutten et al. (2023) [52]				
77 ± 5 years (68–89) *n* = 109 39% male	Inclusion Age > 65 years Clinical DRS = 0 MMSE > 25 Delayed Recall of Logical Memory Story A > cutoff adjusted by age and education Geriatric Depression Scale < 11 Exclusion History of alcohol use History of drug use History of head trauma Current serious medical or psychiatric illness	Global [Preclinical Alzheimer’s Cognitive Composite-5, including MMSE, WMS-R (Logical), DSCT, free and cued selective reminding test, and CVFT] Processing Speed [TMT-A, DSCT] Executive Function [Controlled Oral Word Association Test, TMT-B/A] Memory [WMS-R (Logical), selective reminding test, free and cued selective reminding test]	**Touch screen tap**^CBB^ Simple RT (detection) 1-choice task Choice RT (identification) 2 choices Feature: color Complex RT Stimulus: playing cards Determine if you have seen the stimulus before (one-card learning). Determine if the stimulus is the same as the previous (one back).	Intraindividual variability in RT was measured each month for one year. An association between cognitive tests and RT was not assessed for a single session. Greater variability in combined simple and choice RT was associated with poorer baseline performance in global cognition, processing speed, and memory without adjustment for mean RT. Greater variability in complex RT was associated with poorer baseline performance in executive function with and without adjustment for mean RT.
Vasquez et al. (2016) [65]				
74 ± 6 years (65–85) *n* = 48 ^∧^ 27% male	Inclusion Normal cognitive function assessed by the modified TICS Exclusion History of significant head injury Neurological or major medical illness Radiation to the head Drug abuse Current use of psychiatric medications Not fluent in English	Global [MMSE, WAIS-III] Processing Speed [WAIS-III (Digit Symbol Coding)] Executive Function [WCST, D-KEFS (Trail Making and Color-Word Interference)] Learning and Memory [WMS-III (Logical Memory and Digit Span ^h^ forwards and backwards)] Language [BNT, D-KEFS (Fluency ^h^)]	**Screen tap with a stylus** Complex RT Playing cards moved horizontally across the screen Target: 8 of spades Features: color, suit, and number; distractor cards shared 0, 1, or 2 features with the target	When combined across young and old adults, age-related differences in RT distribution were associated with executive function. • WCST, D-KEFS Trail Making, and D-KEFS Color-Word Interference had the strongest associations. • WMS-III Digit Span and D-KEFS Fluency were weakly associated. The associations of global cognition and processing speed with RT were not assessed. The associations of executive function, learning and memory, and language with RT were not assessed directly.

Study populations are reported as average ± standard deviation (range) based on available data. ^ Study population reported for older adult group without cognitive impairment; CBBCogstate Brief Battery tests; ^a^ Working Memory; ^b^ Fluidity; ^c^ Auditory Attention; ^d^ Sustained Attention; ^e^ Episodic Memory; ^f^ Semantic Memory; ^g^ Reasoning; ^h^ Authors classified as executive function domain; ADLs: Activities of Daily Living; BDS: Blessed Dementia Scale; BNT: Boston Naming Test; CVFT: Category Verbal Fluency Test; CVLT: California Verbal Learning Test; D-KEFS: Delis-Kaplan Executive Function System; DRS: Dementia Rating Scale; DSCT: Digit Symbol Coding Test; IADLs: Instrumental Activities of Daily Living; MCI: Mild Cognitive Impairment; MMSE: Mini-Mental State Examination; MoCA: Montreal Cognitive Assessment; RT: Reaction Time; TICS: Telephone Inventory of Cognitive Status; TMT: Trail Making Test; WAIS-R, WAIS-III: Wechsler Adult Intelligence Scale—Revised or 3rd edition; WCST: Wisconsin Card Sorting Test; WMS-R, WMS-III: Wechsler Memory Scale—Revised or 3rd edition. The subtitles for (A) and (B) are shaded grey. Upper extremity movements are indicated in bold font and inclusion/exclusion criteria, cognitive domains and subdomains, and types of RT measures are underlined.

**Table 3:** Summary matrix of evidence strength for the association between cognition and RT in older adults. Strength is mapped by cognitive domain and type of RT measures. Moderate: consistent association (≥4 studies); Weak: limited (<4 studies) or mixed association; None: no associations reported.

		RT Measure		
		Simple	Choice	Complex
Cognitive Domain—Subdomain	Global Cognition	None	Weak	None
	Sensation and Perception	None	None	None
	Motor Skills and Construction	None	None	None
	Perceptual Motor Function —Processing/ Perceptual Speed	None	Weak	Weak
	—Visuospatial Ability	None	None	None
	Executive Function	None	Weak	Weak
	Attention	None	Weak	Weak
	Learning and Memory —Learning	None	None	Weak
	—Memory	None	Moderate	Weak
	Language	None	None	Weak

## Data Availability

Data is contained within the article or Supplementary Materials.

## Supplementary Materials

The following supporting information can be downloaded at: https://www.mdpi.com/article/10.3390/neurosci6030071/s1, Table S1: Studies that measured cognitive function and RTs in older adults but did not assess the association between measures; Table S2: Direct or indirect association between cognitive tests and choice or complex RT in older adults.

## Abbreviations

The following abbreviations are used in this manuscript:

RT	Reaction Time
PEDro	Physiotherapy Evidence Database.
PRISMA	Preferred Reporting Items for Systematic reviews and Meta-Analyses
MCI	Mild cognitive impairment
DRS	Dementia Rating Scale
ADL	Activities of Daily Living
IADL	Instrumental Activities of Daily Living
MoCA	Montreal Cognitive Assessment
TMT	Trail Making Test
MMSE	Mini-Mental State Examination
BDS	Blessed Dementia Scale
WAIS-R	Wechsler Adult Intelligence Scale—Revised
WCST	Wisconsin Card Sorting Test
CVLT	California Verbal Learning Test
WMS-R	Wechsler Memory Scale—Revised
BNT	Boston Naming Test
WMS	Weschler Memory Scale
WAIS-III	Wechsler Adult Intelligence Scale—3rd edition
DSCT	Digit Symbol Coding Test
CVFT	Category Verbal Fluency Test
TICS	Telephone Inventory of Cognitive Status
D-KEFS	Delis-Kaplan Executive Function System
WMS-III	Weschler Memory Scale—3rd edition
CBB	Cogstate Brief Battery
EEG	Electroencephalogram
ROCFT	Rey-Osterrieth Complex Figure Test
SCWT	Stroop Color and Word Test
SDMT	Symbol Digit Modalities Test
AVLT	Auditory Verbal Learning Test
COMP	CompCog computerized cognitive screening battery
Benton JoLO	Benton Judgement of Line Orientation
HVLT-R	Hopkins Verbal Learning Test—Revised
BVMT-R	Brief Visuospatial Memory Test—Revised
WMS-IV	Wechsler Memory Scale—4th edition
VTS	Vienna Test System
SPMSQ	Short Portable Mental Screening Questionnaire
WAIS-IV	Wechsler Adult Intelligence Scale—4th edition
PACC5	Preclinical Alzheimer’s Cognitive Composite-5
COWAT	Controlled Oral Word Association Test
INT	Internally driven uncertainty
EXT	Externally cued uncertainty
STM	CompCog Visual and Spatial Short Term Memory subtest
