# Supplementary material for: Limited Evidence to Review—Is There an Association Between Cognition and Upper Extremity Motor Reaction Time in Older Adults?
Source: NeuroSci. 2025 Jul 30;6(3):71. doi: 10.3390/neurosci6030071 (PMC12372119; doi:10.3390/neurosci6030071)
Supplement: Supplementary file 1 [file neurosci-06-00071-s001.zip › neurosci-3701438-supplementary.pdf]

Supplementary Materials:

Table S1. Studies that measured cognitive function and RTs in older adults but did not assess the association between measures.

Study Population	Inclusion and Exclusion Criteria	Cognitive Domain or Subdomain [test]	Movement and RT Measures	Results and Limitations
**Chen et al. (2020)** [45]				
68±4 years (62–76)<br>n=28^<br>54% male	Inclusion<br>Normal cognitive function in five domains assessed<br>Exclusion<br>MMSE < 24 (Chinese version)<br>Impaired ADLs<br>Impaired IADLs<br>Low accuracy in the task<br>Poor EEG signal quality	Global<br>[MMSE]<br>Visuospatial Ability<br>[ROCFT (Copy Test), Clock Drawing Test]<br>Executive Function<br>[TMT-B, SCWT]<br>Attention<br>[TMT-A, SDMT]<br>Memory<br>[AVLT (Delayed Recall & Total), ROCFT (Recall Test)]<br>Language<br>[CVFT, BNT]	**Mouse button press**<br>Complex RT<br>Arrow direction (left or right)<br>Congruent or incongruent with row of arrows	Electroencephalography measured neural signals related to preparatory attention.<br>• For the congruent condition, the relationship between neural measures of preparatory attention and RT was associated with memory.<br>• For the incongruent condition, the relationship between neural measures of preparatory attention and RT was not associated with cognition.
**Ferreira et al. (2022)** [46]				
72±5 years<br>n=27<br>67% male	Inclusion<br>Community living<br>No contraindications to moderate aerobic exercise<br>Age > 60 years<br>Exclusion<br>MMSE ≤ cutoff adjusted by education level	Processing Speed<br>[TMT-A, TMT-B]<br>Language<br>[phonemic and semantic verbal fluency[a]]	**Key press**<br>Simple RT<br>1-choice task<br>Choice RT<br>4 choices<br>Feature: location	Exercise condition did not influence cognitive performance or RT measures.
**Hartle et al. (2022)** [47]				
71±6 years (60–85)<br>n=28^<br>18% male	Exclusion<br>Condition affecting cognition<br>Recent history of alcohol or drug dependence<br>High levels of depressive symptoms<br>Uncorrected vision or hearing disorder	Global<br>[MMSE]<br>Motor Skills & Construction<br>[Clock Drawing Test]<br>Processing Speed<br>[Simple RT, Choice RT]<br>Attention	**Touch screen button press**[COMP]<br>Simple RT<br>1-choice task<br>Choice RT<br>2 choices<br>Feature: color	Processing speed was measured from motor RT tasks.<br>RT during cognitive domain tests of attention and memory was able to identify mild cognitive impairment in older adults.

	Illiteracy<br>Medication that could affect RT	[Inhibitory Control Test^{COMP}, Stroop Test^{COMP}, Survey Test^{COMP}]<br>Learning & Memory<br>[Figure Memory Test, Implicit Learning Test^{COMP}, Visual and Spatial Short-Term Memory$^{\mathrm{COMP,b}}$, Face Recognition and Memory$^{\mathrm{COMP,c}}$]<br>Language<br>[CVFT]		
**Hennessy et al. (2025)** [48]				
67±5 years<br>(60–77)<br>n=25^<br>40% male	Inclusion<br>60–90 years<br>English speaking<br>Able to travel for in-person testing<br>Normal or corrected to normal vision<br>Exclusion<br>Previous or current neurological disorder diagnosis<br>Current diagnosis of dementia<br>Severe psychiatric illness	Motor Construction<br>[Rey Complex Figure Test]<br>Processing Speed<br>[TMT-A, TMT-B, Symbol Digit Modalities Test]<br>Attention<br>[TMT-A, TMT-B]<br>Learning & Memory<br>[Digit Span Test, Hopkins Verbal Learning Test–Revised, Rey Complex Figure Test]	**Key press**<br>Simple RT (detection)<br>1-choice task<br>Choice RT (discrimination)<br>2 choices<br>Feature: orientation of sinusoidal grating<br>Complex RT (Symmetrical Outcome-Revaluation Task)<br>8 stimulus-outcome associations<br>Go/NoGo response valuation and change	Choice RT but not simple RT was longer in older adults.<br><br>Processing speed was measured from detection and discrimination RT tasks as well as cognitive tests of processed speed. Processing speed was slower in older adults.
**Hong et al. (2020)** [49]				
68±6 years<br>n=22^<br>36% male	Inclusion<br>Neurologically and psychiatrically healthy<br>MMSE ≥ 24<br>Normative scores in all or all but one domain of a comprehensive neuropsychological evaluation<br>Exclusion	Sensation & Perception<br>[Benton JoLO]<br>Processing Speed<br>[SDMT]<br>Executive Function<br>[TMT-B, SCWT]<br>Attention<br>[WAIS-IV (Digit Span), TMT-A]<br>Learning	**Button press**<br>Choice RT<br>4 choices<br>Feature: location, repeated or random sequence	Cognitive tests other than learning were used to group participants by presence of cognitive impairment.<br><br>Implicit learning was measured by the change in RT with exposure to repeated target sequences relative to simple motor learning which was measured by the change in RT for random target sequences.

	Significant acute or chronic medical conditions<br>Severe psychiatric or neurological history<br>Current psychotropic substance use<br>Medications for significant medical conditions	[Implicit learning]<br>Memory<br>[HVLT-R (Immediate & Delayed Recall), BVMT-R (Immediate & Delayed Recall), WMS-IV (Logical Memory I & II)]<br>Language<br>[D-KEFS (Letter & Category Fluency)]		Older adults demonstrated implicit learning in later stages of the task.
**Jardim et al. (2024)** [50]				
71±0 years (60–93)<br>n=236^<br>23% male	Inclusion<br>MMSE > cutoff adjusted by education level<br>Snellen visual acuity 20/30<br>No history of traumatic brain injury, stroke or depression	Attention<br>[Motor Screening Task[d], Rapid Visual Processing[e]]<br>Memory<br>[Paired Associates Learning[c], Spatial Working Memory[b]]	**Button release**<br>Simple RT<br>1-choice task<br>Choice RT<br>5 choices<br>Feature: location	Episodic memory, sustained attention and simple RT task accuracy contributed to grouping older adults by cognitive function but working memory did not.<br><br>Choice RT was not reported.
**Kitchen and Miall (2019)** [54]				
71±5 years (>65)<br>n=31^<br>35% male	Inclusion<br>Right hand dominant<br>MoCA ≥ 26<br>Exclusion<br>History of neurological illness<br>Pain or limitation of the arm, wrist or fingers	Sensation & Perception<br>[Proprioception measured as bias and uncertainty from error in manipulated planar reaching without visual feedback]<br>Memory[b]<br>[Backward Digit Span Test]	**Planar reaching**<br>Choice RT<br>3 choices<br>Feature: location	There was no association between working memory and proprioception in older adults.
**Krumpolt et al. (2025)** [56]				
67±1 years (65–69)<br>n=60<br>50% male	Inclusion<br>Physically and mentally unimpaired<br>No regular sporting activity for ≥ 2 years<br>No cognitive impairment as assessed by MMSE	Processing Speed<br>[Simple RT, Choice RT]<br>Executive Function<br>[Stroop task[VTS,f]]	**Button press or release**[VTS]<br>Simple RT<br>1-choice task<br>Choice RT<br>2 choices<br>Feature: color	RT was measured as a cognitive test and not assessed for an association with other cognitive tests.<br><br>Sex affected performance on cognitive tests and the effect of exercise intervention.
**Mack et al. (2025)** [57]				

69±4 years<br>(64–78)<br>n=153<br>42% male	Inclusion<br>Right hand dominant<br>Retired<br>Fluent French or German<br>General good health<br>No history of regular musical practice over the lifespan<br>Exclusion<br>Uncorrected auditory or visual impairment<br>Neurological disease<br>Cognitive impairment<br>Cardiovascular disease	Global<br>[Cognitive Telephone Screening Instrument]	**Mouse button press**<br>Complex RT<br>**Number Switch Test:**<br>Digits in various colors<br>Feature: magnitude (low or high), parity (even or odd), or mixed<br>**Perceptual Switch Test:**<br>Letters in blue or orange<br>Feature: capital (lower or upper), color (blue or orange), or mixed	RT was dependent on cognitive processes and not assessed for an association with global cognition.<br><br>Greater RT performance improvement was correlated with lower initial cognitive function.<br><br>RT costs of switching and mixing tasks differed for perceptual switch and number switch tests.
**Qiu and Xiong (2017)** [58]				
72±5 years<br>(60–80)<br>n=80<br>0% male	Inclusion<br>Non-fallers<br>Exclusion<br>Male<br>Neurological and musculoskeletal diseases or vestibular dysfunction	Processing Speed<br>[TMT-A, TMT-B]<br>Executive Function<br>[TMT-B]	**Right hand button tapping on an iPad**<br>Simple RT<br>1-choice task<br>Choice RT<br>2, 4, 10 choices<br>Feature: location	An association between cognitive tests and RT was not assessed.
**Rattanavichit et al. (2022)** [59]				
69±6 years<br>(60–85)<br>n=60^<br>40% male	Inclusion<br>Able to communicate<br>No visual or hearing impairment<br>MoCA ≥ 25 (Thai version)<br>Exclusion<br>Depression or psychological condition<br>History of movement disorder<br>Unstable diabetes mellitus<br>Alcohol use disorder<br>Cigarette smoking > 10 pack years	Global<br>[MoCA]<br>Perceptual Motor Function<br>[clock reading test, stick design test, stick catching test]<br>Executive Function<br>[Digit Span Test, SCWT, modified Switching Verbal Fluency Test]	**Stick catching with pincer grip**<br>Simple RT<br>1-choice task	There was no age-related difference in RT.<br><br>Older adults performed poorer in cognitive tests of executive function and perceptual motor function except for the simple RT stick catching task.
**Sleimen-Malkoun et al. (2013)** [60]				

78±7 years<br>n=14<br>50% male	<u>Inclusion</u><br>Independent<br>Physically active<br>Naïve to experiment<br><u>Exclusion</u><br>Cognitive or motor pathology<br>Uncorrected vision loss<br>Depression	<u>Motor Skills & Construction</u><br>[Clock Drawing Test]<br><u>Processing Speed</u><br>[Computed as change in measured response time with change in level of task difficulty]	**Right hand target aiming**<br><u>Complex RT</u><br>Difficulty scaled with target distance<br>**Right index finger button press**<br><u>Simple RT</u><br>1-choice task<br><u>Choice RT</u><br>Difficulty scaled with choices: 2, 4, 8, 16<br>Feature: location, incompatible	The Clock Drawing Test was used for study inclusion.<br><br>Processing speed was measured from motor (target aiming) and cognitive (button press) RT tasks.<br><br>Processing speed was slower for older adults, especially with higher levels of difficulty, but did not differ between tasks.
**Tait et al. (2024)** [62				
77±7 years (65–96)<br>n=299[v]<br>27% male	<u>Inclusion</u><br>Age > 65 years<br>Fall risk<br>No cognitive impairment on SPMSQ<br>English proficiency<br>Able to walk 50 m with no or minimal assistance<br><u>Exclusion</u><br>≥ 150 min of moderate-vigorous activity/week<br>Resistance or balance training in past 3 months<br>Musculoskeletal or neurological disease or acute/terminal illness<br>Unstable cardiovascular or respiratory disorder<br>Fracture in past 3 months<br>Uncorrected visual impairment	<u>Processing Speed</u>[f]<br>[Simple RT, Choice RT]<br><u>Executive Function</u><br>[Groton Maze Learning Test [CBB], One Back Test [CBB,b]]<br><u>Learning & Memory</u><br>[One Card Learning Test [CBB]]	**Touch screen button press** [CBB]<br><u>Simple RT</u> (detection)<br>1-choice task<br><u>Choice RT</u> (identification)<br>2 choices<br>Feature: color	Processing speed was measured from motor RT tasks.<br><br>Participation in a dual-task, functional power training program for six (6) months improved simple and choice RT measures but did not affect other measures of cognition.
**Unger et al. (2025)** [63]				

70±4 years (62–79)<br>n=34^<br>56% male	Inclusion<br>Normal or corrected-to-normal vision<br>Exclusion<br>Affective disorder<br>Neurological disease<br>Psychotropic mediation<br>Color blindness	Processing Speed<br>[Digit Symbol Substitution]<br>Executive Function<br>[Raven's Advanced Progressive Matrices, modified Digit-Ordering Test[b]]<br>Language<br>[Spot-the-Word Test]	**Key press**<br>Choice RT<br>2 choices<br>Feature: food type or size<br>Complex RT<br>Switch between choice features every 2nd trial	A condition designed to increase positive affect increased RT and accuracy for older adults on trials requiring a switch between tasks but did not influence RT or accuracy for mixed task trials.
**Van Humbeeck et al. (2024)** [64]				
73±4 years (66–82)<br>n=37^<br>43% male	Exclusion<br>Recent fall or sports accident<br>History of neurological disorders or stroke<br>Neurodevelopmental disorder affecting balance<br>Cognitive impairment<br>Acute musculoskeletal injury<br>Alcohol consumption < 24 hours prior	Learning & Memory<br>[Digit Span, Digit symbol substitution, visuo-spatial working memory]	**Mouse button click**<br>Simple RT<br>1-choice task	Dual performance of visuo-spatial working memory and simple RT tasks resulted in longer RTs in older adults.<br><br>Independent measures of cognitive and motor performance were not assessed for an association.
**Worschech et al. (2024)** [67]				
73±4 years<br>n=86<br>48% male	Inclusion<br>Healthy<br>No signs of neurological, psychological or motor problems<br>Exclusion<br>Participants with extensive piano experience	Motor Skills & Construction<br>[Purdue Pegboard, Clicking speed]<br>Processing Speed<br>[Number Connections Test, TMT-A, WAIS-IV (Coding, Symbol Search), Digit Span]<br>Memory[b]<br>[Reading letters, TMT-B, Digit Span, Corsi blocks]	**Key press**<br>Complex RT<br>Motor Sequence (piano-related and unrelated)<br>8 choices<br>Feature: location, color	RT was not reported.<br><br>Learning rate was estimated from performance based on errors.<br><br>Motor sequence performance was associated with processing speed for piano-related and unrelated tasks.<br><br>Motor sequence performance was associated with working memory only for the piano-related task.

Study populations reported as average ± standard deviation (range) based on available data. ^Study population reported for older adult group without cognitive impairment, ˅Less than 10% of study group classified as having mild cognitive impairment after testing. [COMP]Subtests of CompCog

computerized cognitive screening battery, [CBB]Cogstate Brief Battery tests, [VTS]Subtests of Vienna Test System. [a]Authors classified as executive function domain, [b]Working Memory, [c]Episodic Memory, [d]Also classified as processing speed, [e]Sustained Attention, [f]Also classified as attention AVLT: Auditory Verbal Learning Test; Benton JoLO: Benton Judgement of Line Orientation; BNT: Boston Naming Test; BVMT-R: Brief Visuospatial Memory Test – Revised; CVFT: Category Verbal Fluency Test; DRS: Dementia Rating Scale; D-KEFS: Delis-Kaplan Executive Function System; HVLT-R: Hopkins Verbal Learning Test - Revised; MMSE: Mini-Mental State Examination; MoCA: Montreal Cognitive Assessment; RT: Reaction Time; ROCFT: Rey-Osterrieth Complex Figure Test; SCWT: Stroop Color and Word Test; SDMT: Symbol Digit Modalities Test; SPMSQ: Short Portable Mental Screening Questionnaire; TMT: Trail Making Test; WAIS-III WAIS-IV: Wechsler Adult Intelligence Scale – 3rd or 4th edition; WCST: Wisconsin Card Sorting Test; WMS-R: Wechsler Memory Scale – Revised edition. Upper extremity movements are indicated in bold font and inclusion/exclusion criteria, cognitive domains and subdomains, and types of RT measures are underlined.

Table S2. Direct or indirect association between cognitive tests and choice or complex RT in older adults

Cognitive Domain – Subdomain				
**Cognitive Test**	**Study**	**Association** ☑	☐	☒
**Global Cognition**				
COGTEL	[57]		☐	
Dementia Scales (e.g. BDS, DRS)	[55]		☐	
MMSE	[45, 55, 57, 65]		☐	
MoCA	[53]	☑		
	[59]		☐	
PACC5	[52]	☑		
WAIS-III	[65]		☐	
**Sensation & Perception**				
Benton JoLO	[49]		☐	
Proprioception	[54]		☐	
**Motor Skills & Construction**				
Clicking Speed	[67]		☐	
Clock Drawing Test	[47, 60]		☐	
Purdue Pegboard	[67]		☐	
Rey Complex Figure	[48]		☐	
**Perceptual Motor Function**				
**– Processing/Perceptual Speed**				
Digit Span	[66]	☑		
	[67]		☐	
Digit Symbol Substitution	[63]		☐	
DSCT	[52]	☑		
Motor task, varied difficulty	[47, 50, 56, 60, 62]		☐	
Number Connection Test	[67]		☐	
Number Symbol Test	[66]	☑		
SDMT	[48, 49]		☐	
TMT-A	[52, 53, 68]	☑		
	[46, 48, 55, 58, 67]		☐	
TMT-B	[68]	☑		
	[46, 48, 58]		☐	
	[53]			☒
WAIS-R, -III, -IV (Digit Symbol Coding, Digit Symbol Modalities, Symbol Search)	[55, 65, 67]		☐	
**– Visuospatial Ability**				
Clock Drawing Test	[45]		☐	
Clock Reading Test	[59]		☐	
ROCFT (Copy Test)	[45]		☐	
Stick Design Test	[59]		☐	
WAIS-R (Block Design)	[55]		☐	
**Executive Function**				
Clock Drawing Test	[55]		☐	
COWAT	[52]	☑		
Digit Span	[59]		☐	
DKEFS (Color-Word Interference & Trail Making)	[65]	☑		
Groton Maze Learning Test	[62]		☐	
Letter Fluency	[55]int	☑		
	[55]ext			☒
Modified Digit-Ordering Test	[63]		☐	
One Back Test	[62]		☐	
Raven's Advanced Progressive Matrices	[66]		☐	
SCWT or Stroop Task	[45, 49, 56, 59]		☐	
TMT-B	[52], [55]int	☑		
	[45, 49, 58]		☐	
	[53][65]ext			☒
Verbal Fluency	[59]		☐	
WAIS-III	[68]	☑		
WCST (Modified)	[55]int, [65]	☑		
	[55]ext			☒
WMS-III (Digit Span)	[65]	☑		
**Attention**				
Backwards Counting	[44]	☑		
CVOE Switch	[66]	☑		
Inhibitory Control Test	[47]		☐	
Rapid Visual Processing	[50]		☐	
SDMT	[45]		☐	
Stroop Test	[66]	☑		
	[47]		☐	
Survey Test	[47]		☐	
Sustained Attention to Response Task	[61]		☐	
TMT-A	[53]	☑		
	[45, 48, 49]		☐	
TMT-B	[48]		☐	
WAIS-R, -IV (Digit Span)	[49, 55]		☐	
**Learning & Memory**				
**– Learning**				

Digit Symbol Substitution	[64]		☐	
General Skill Learning	[51]	☑		
Implicit Learning	[47, 49]		☐	
Triplet Learning	[51]			☒
**– Memory**				
AVLT	[45]		☐	
Corsi Blocks	[67]		☐	
CVLT	[55]$_{ext}$	☑		
	[55]$_{int}$			☒
Craft Story 21 (Immediate & Delayed Recall)	[66]	☑		
Digit Span	[48, 54, 59, 64, 67]		☐	
Face Recognition and Memory	[47]		☐	
Figure Memory Test	[47]		☐	
HVLT-R	[48, 49]		☐	
Immediate Free Recall	[68]	☑		
One Card Learning Test	[62]		☐	
Paired Associates Learning	[50]		☐	
Reading letters	[67]		☐	
Rey Complex Figure	[48]		☐	
ROCFT (Recall Test)	[45]		☐	
Selective Reminding Tests	[52, 66]	☑		
Spatial Working Memory	[50]		☐	
TMT-B	[67]		☐	
Visuospatial Memory (e.g. BVMT-R, STM)	[47, 49, 64]		☐	
WMS (Paired Associative Recall)	[66]	☑		
WMS-R (Visual Reproduction & Logical Memory)	[55]$_{ext}$ [52]	☑		
	[55]$_{int}$			☒
WMS-III (Digit Span)*	[65]	☑		
WMS-III, -IV (Logical, I&II)	[49, 65]		☐	
**Language**				
BNT	[45, 55, 65]		☐	
CVFT	[66]	☑		
	[45, 47]		☐	
DKEFS (Fluency)*	[65, 66]	☑		
	[49]		☐	
Fluency	[53]**	☑		
	[46]		☐	
Multilingual Naming Test	[66]	☑		
Spot-the-Word Test	[63]		☐	
Vocabulary	[68]			☒
WAIS-R (Fluency)	[55]		☐	

☑ Association between cognitive test and RT
☐ Cognitive test performance not reported or reported but not compared to RT
☒ No association between cognitive test and RT

$_{int}$Internally driven uncertainty, $_{ext}$Externally cued uncertainty, *Some authors classified as executive function domain. **Authors referred to as fluidity. AVLT: Auditory Verbal Learning Test; Benton JoLO: Benton Judgement of Line Orientation; BDS: Blessed Dementia Scale; BNT: Boston Naming Test; BVMT-R: Brief Visuospatial Memory Test – Revised; COGTEL: Cognitive Telephone Screening Instrument; COWAT: Controlled Oral Word Association Test; CVFT: Category Verbal Fluency Test; CVLT: California Verbal Learning Test; DKEFS: Delis Kaplan Executive Function System; DRS: Dementia Rating Scale; DSCT: Digit Symbol Coding Test; HVLT-R: Hopkins Verbal Learning Test - Revised; MMSE: Mini-Mental State Examination; MoCA: Montreal Cognitive Assessment; PACC5: Preclinical Alzheimer's Cognitive Composite-5; RT: Reaction Time; ROCFT: Rey-Osterrieth Complex Figure Test; SCWT: Stroop Color and Word Test; SDMT: Symbol Digit Modalities Test; STM: CompCog Visual and Spatial Short-Term Memory subtest; TMT: Trail Making Test; VTS: Vienna Test System; WAIS-R WAIS-III WAIS-IV: Wechsler Adult Intelligence Scale – Revised, 3rd or 4th edition; WCST: Wisconsin Card Sorting Test; WMS-R WMS-III WMS-IV: Wechsler Memory Scale – Revised, 3rd or 4th edition
